# Iron uptake and transport across physiological barriers

**DOI:** 10.1007/s10534-016-9952-2

**Published:** 2016-07-25

**Authors:** Kari A. Duck, James R. Connor

**Affiliations:** 1 Department of Neurosurgery, The Pennsylvania State University College of Medicine, Hershey, PA USA; 2 Department of Neurosurgery, Neural and Behavioral Sciences and Pediatrics, Center for Aging and Neurodegenerative Diseases, Penn State Hershey Medical Center, 500 University Drive, MC H110, C3830, Hershey, PA 17033 USA

**Keywords:** Iron transport, Gut, Placenta, Blood–brain barrier, HFE

## Abstract

Iron is an essential element for human development. It is a major requirement for cellular processes such as oxygen transport, energy metabolism, neurotransmitter synthesis, and myelin synthesis. Despite its crucial role in these processes, iron in the ferric form can also produce toxic reactive oxygen species. The duality of iron’s function highlights the importance of maintaining a strict balance of iron levels in the body. As a result, organisms have developed elegant mechanisms of iron uptake, transport, and storage. This review will focus on the mechanisms that have evolved at physiological barriers, such as the intestine, the placenta, and the blood–brain barrier (BBB), where iron must be transported. Much has been written about the processes for iron transport across the intestine and the placenta, but less is known about iron transport mechanisms at the BBB. In this review, we compare the established pathways at the intestine and the placenta as well as describe what is currently known about iron transport at the BBB and how brain iron uptake correlates with processes at these other physiological barriers.

## Introduction

Iron (Fe) is an essential, multifunctional micronutrient that has been shown to function in oxygen transport through its role in hemoglobin, as a crucial cofactor in the electron transport chain, and as a cofactor during DNA synthesis (Beard et al. 1996). The multifunctionality of iron stems from its ability to easily transition between its two oxidation states, ferrous (Fe^2+^) and ferric (Fe^3+^). The process by which iron transitions between states is known as Fenton chemistry represented as:

1 $${\text{Fe}}^{2 + } + {\text{ H}}_{ 2} {\text{O}}_{ 2} \to {\text{ Fe}}^{3 + } + {{\text{ HO}} ^\cdot} \, + {\text{ OH}}^{ - }$$

2 $${\text{Fe}}^{3 + } + {\text{ H}}_{ 2} {\text{O}}_{ 2} \to {\text{ Fe}}^{2 + } + {{\text{ HOO}}^\cdot} \, + {\text{ H}}^{ + }$$

During this reaction, ferrous iron reacts with hydrogen peroxide to generate a hydroxyl radical and ferric iron reacts with hydrogen peroxide to form a hydroperoxyl radical (Chevion 1988; Stohs and Bagchi 1995).

Iron imbalance, whether too much or too little, can be harmful. Too little iron can cause iron deficiency with subsequent anemia, which is the most common nutrient disorder worldwide (Iron Deficiency Anemia 2016). The condition presents with decreased red blood cell production and reduced hemoglobin levels and is associated with fatigue and weakness in patients. While having too little iron can have negative effects, too much iron can also be harmful. When iron participates in Fenton chemistry, it generates toxic free radicals and reactive oxygen species, which can be detrimental to cell health leading to damage of lipids, proteins, and DNA, ultimately resulting in cell death. Thus, it is crucial for iron levels to be tightly regulated. Physiological levels of iron typically remain constant due to a minimal excretion mechanism so homeostasis is maintained by strict regulation of iron uptake at the gut. During pregnancy, it is important to maintain iron homeostasis in not only the mother, but also in the fetus. The placenta acts as a barrier at which iron transfer can be regulated by both the mother and the fetus. Similarly, the brain also must be protected from iron imbalances because its high rate of oxygen consumption results in high iron requirements yet its concentration of lipids makes it particularly vulnerable to oxidative damage (Raichle and Gusnard 2002; Sokoloff et al. 1955). To access the brain, iron must be transported across the blood–brain barrier (BBB). This barrier is composed of the brain microvasculature, which is distinct from other microvessels in the body because it forms tight junctions, blocking passive diffusion between cells into the brain. While iron transfer at both the gut and the placenta have been studied for decades, research into the mechanisms and regulation at the BBB are less understood. In this review, we compare and contrast mechanisms and regulation of iron transport at the gut, placenta, and BBB as an attempt to further elucidate important factors to be studied in the BBB model.

## Iron transport in the duodenum

**Fig. 1:**
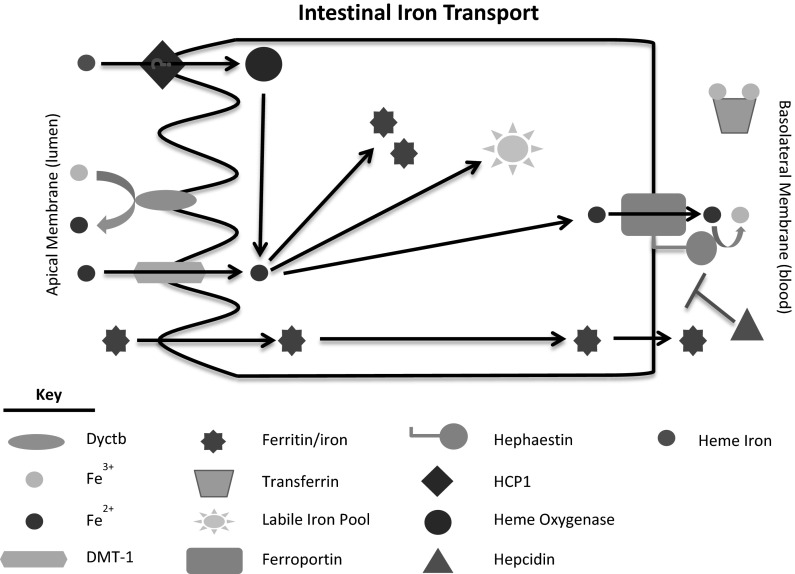
Schematic of intestinal iron transport. Briefly, the primary mechanism by which iron is taken up by the enterocyte is through DMT-1 on the luminal membrane after reduction by Dyctb. Iron has also been suggested to be transported as heme through HCP1 and as ferritin, but these mechanisms are controversial (heme) or less investigated (ferritin). Once in the intracellular labile iron pool, the iron can be stored in ferritin or exported into the body circulation through ferroportin. The ferroxidase, hephaestin, converts the ferrous iron that is released to ferric iron for use by transferrin

The gut functions as the key modulator of iron concentration in the body. A sophisticated mechanism of intestinal uptake and regulation has evolved so that sufficient quantities of iron are absorbed to fulfill daily requirements and maintain sufficient iron stores while not allowing for excessive amounts of iron to accumulate (Fig. 1). The importance of the gut’s role in maintaining iron homeostasis through absorption stems from the body’s lack of an iron excretion mechanism. Skin exfoliation, sloughing of intestinal epithelium, and menstruation remain the three primary processes by which iron is excreted, as very little is removed from the body in urine or feces (Cole et al. 1971; Finch et al. 1970; Green et al. 1968). On average, the body loses only 1–2 mg of iron/day (Cole et al. 1971; Finch et al. 1970; Green et al. 1968). At the same time, the body uses 20–25 mg of iron/day, most of which is required for erythropoiesis (Bothwell et al. 1958a; Hentze et al. 2004). The majority of the body’s iron is stored to address both acute and chronic iron needs, making regulation of absorption at the the gut crucial so that the body’s iron needs are met without depleting stores. Discussed in more detail below, the enterocytes of the intestines are renewed regularly from a population of crypt cells that are exposed to systemic iron and alter their iron management protein profiles accordingly to alter the amount of iron transport they will support.

### Mechanism of iron transport in the duodenum

The mechanism by which iron moves from the intestinal lumen into the bloodstream occurs in three stages—(1) luminal uptake and transport across the apical membrane, (2) transfer to the basolateral membrane, and (3) transport across the basolateral membrane into the circulation. In the first stage of transport, iron must cross from the lumen of the intestine and into the enterocyte. The first mechanism by which iron gets into the enterocyte requires that ferric iron be reduced by duodenal cytochrome B (DcytB) to its ferrous form (Luo et al. 2014; McKie et al. 2001; Vlachodimitropoulou et al. 2010). The ferrous iron can then be transported through divalent metal transporter 1 (DMT-1), a known mediator of cationic metal transport that is present at the apical membrane of the enterocyte (Canonne-Hergaux et al. 1999; Gunshin et al. 1997; Mackenzie and Garrick 2005). Heme iron is imported by the enterocyte, but the mechanism remains undetermined. Heme carrier protein 1 (HCP1) was identified in the enterocyte and later shown to function as a low-affinity heme transporter into the enterocyte (Le Blanc et al. 2012; Shayeghi et al. 2005). HCP1’s role as the primary mode of heme uptake remains in question, however, as research has implicated this protein in folate transport rather than in heme transport (Qiu et al. 2006). Any heme that may be taken up by the cell is catabolized by heme oxygenase (HO), causing the release of ferrous iron into the cell’s labile iron pool (Raffin et al. 1974; West and Oates 2008). Another potential direct source of iron is ferritin which can traverse the enterocyte and accumulate in the blood, but the pathway by which this occurs remains unstudied (Williams and Hemmings 1978).

Upon entering the enterocyte, iron can be transported to and then across the basolateral membrane or it can be taken up by ferritin, an iron storage protein with ferroxidase activity. Ferroportin, an iron transport protein, has been observed on the basolateral membrane of the enterocyte (Donovan et al. 2005). Once at the basolateral membrane, iron passes through ferroportin in its ferrous form. Ferroportin is coupled with hephaestin, a ferroxidase present on the basolateral membrane (Han and Kim 2007; Yeh et al. 2009; Yeh et al. 2011). Hephaestin functions to convert ferrous iron to ferric iron which can then be taken up by apo-Tf in the serum to be transported throughout the body (Chen et al. 2004).

### Regulation of iron transport across the intestinal lumen

The gut has developed an intricate system of checks and balances to regulate iron transport across the enterocyte. The enterocytes of the absorptive intestinal epithelium are the product of crypt cell maturation. Crypt cells have access to the body’s circulation allowing them to take up iron from the blood. When ^59^Fe was injected intravenously, acute accumulation was evident in crypt cells, but not enterocytes (Bedard et al. 1976). The amount of iron accumulation in the crypt cell was dependent on systemic iron status (Bedard et al. 1976). Thus, it was proposed that crypt cells function as a sensor of systemic iron status that can alter their levels of iron management proteins, leading to changes in iron transport when the crypt cells mature into enterocytes. Later studies demonstrated that crypt cells expressed both transferrin (Tf) and transferrin receptors (TfR) that can take up iron from the circulation (Anderson et al. 1994; Levine and Woods 1990). Further characterization of the crypt cells demonstrated ferritin mRNA, but minimal ferritin protein while the mature cells expressed ferritin protein at levels proportional to systemic iron levels (Oates and Morgan 1997). That ferritin mRNA in the crypt cell is not translated until the cell matures provides additional support for the hypothesis that crypt cells can sense systemic iron and alter enterocyte protein expression profile upon maturation into an enterocyte.

The role of the ionic iron transporter, DMT-1, became of interest because studies had shown that crypt cells take up transferrin bound iron from blood while the majority of iron taken up from the intestine by mature enterocytes was non-transferrin bound iron (NTBI) (Waheed et al. 1999). It was shown that in both iron deficiency and in hereditary hemochromatosis, a disease primarily characterized by elevated iron due to a mutation in the HFE protein, both result in iron deficient crypt cells with elevated levels of DMT-1 mRNA (Fleming et al. 1999). The Belgrade rat, which expresses a mutated DMT-1 protein has been used to understand the role of DMT-1 in regulating intestinal iron absorption. These rats have decreased levels of iron and increased DMT-1 gene expression in the enterocyte, as is also seen in the case of iron deficiency where iron absorption across the enterocyte is increased (Knöpfel et al. 2005; Oates et al. 2000). This increase in DMT-1 gene expression indicates that the system is still responsive to the iron deficiency caused by the DMT-1 mutation but that the mutation keeps the Belgrade animal from increasing its iron absorption (Bowen and Morgan 1987; Edwards et al. 1980; Farcich and Morgan 1992; Garrick et al. 1993).

Intestinal iron transport into the body is also regulated at the basolateral membrane of the enterocyte. Here, the enterocyte expresses an iron transporter known as ferroportin (Donovan et al. 2005). Ferroportin is regulated by hepcidin, a peptide that binds to ferroportin and induces internalization of the exporter (Nemeth et al. 2004). Ferroportin is then degraded by the proteasome, reducing iron export. While systemic iron levels can affect uptake into the enterocyte via DMT-1 modulation starting with the crypt cells, they can also affect uptake by adjusting synthesis of hepcidin peptide. When the body has adequate or too much iron, the liver secretes hepcidin, which can then act on the enterocyte to decrease its ferroportin expression and to reduce iron release into the bloodstream. The decrease in iron absorption allows the body to gradually return to normal iron levels and can then be adjusted to maintain normal systemic iron levels. The converse is true for iron deficiency. In this case, the body has relatively low levels of circulating hepcidin allowing ferroportin expression to remain at the basolateral membrane. The enterocytes can continue to release iron until normal systemic iron levels are obtained. As the body attains sufficient amounts of iron, the liver will gradually adjust hepcidin synthesis to maintain normal levels while preventing iron overload.

More recently, H-Ferritin has been implicated in the regulation of iron uptake. Conditional knockdown of H-Ferritin in enterocytes led to a two-fold increase in iron absorption despite iron-overload induced decreases in DMT-1 and Dyctb mRNA levels (Vanoaica et al. 2010). These studies become significant because it implies that the enterocyte is not properly responding to iron absorption cues. The animals exhibited elevated liver iron and, subsequently, elevated hepcidin levels, but enterocyte ferroportin protein was elevated despite no changes in ferroportin mRNA (Vanoaica et al. 2010). Thus, the loss of enterocyte H-Ferritin must override not only the contribution of crypt cells to iron regulation, but also alters the response of ferroportin to hepcidin. These studies demonstrate that intracellular communication is more important in the hierarchy of intestinal iron absorption than current models propose.

## Iron transport across the placenta

The placenta serves as the interface between mother and fetus. It regulates nutrient transport to the fetus including the transport of iron. Of note, iron transfer at the placenta is uni-directional. Once iron is taken up by the placenta, it cannot re-enter the maternal circulation, emphasizing the hierarchy of placental iron transfer (Srai et al. 2002). Evidence shows that maternal iron stores will be depleted before those of the fetus (Gambling et al. 2009). It is also known that the rate of iron transfer across the placenta increases over the course of pregnancy revealing the importance of iron in developmental processes (Davies et al. 1959; Glasser et al. 1968; McArdle et al. 1985a). This increase occurs at a rate not explainable simply by increased transferrin receptors as a result of a larger surface area from placental growth (Jones and Fox 1991). Thus, the changing fetal iron requirements throughout pregnancy suggest the need for a changing placental iron transport mechanics to account for increases in iron transport.

### Mechanism of iron transport at the placenta

**Fig. 2:**
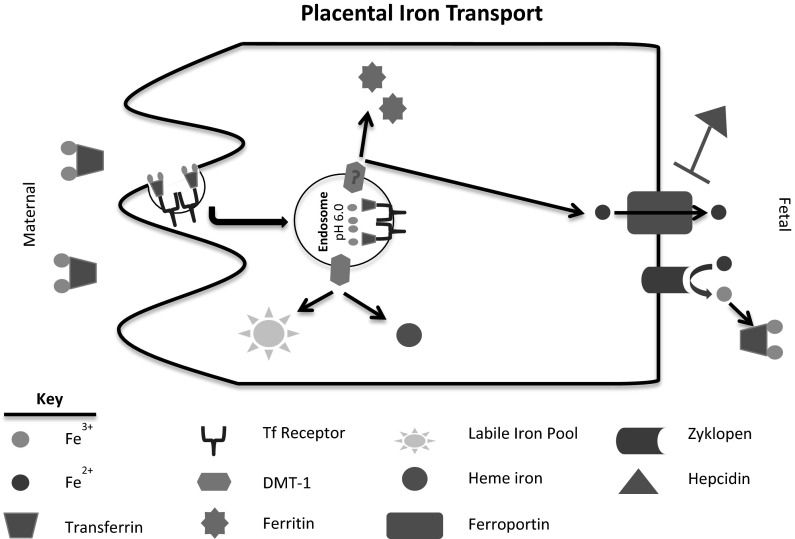
Schematic of placental iron transport. In brief, placental iron transport is completely transferrin-dependent. Transferrin binds to transferrin receptor on the maternal membrane and is endocytosed. The pH decrease in the endosome causes iron to dissociate from transferrin after which it can be transported into the cytoplasm through DMT-1. Once in the cytoplasm, iron can be stored in ferritin, associate with heme, be incorporated into the labile iron pool, or be transported to the fetal circulation through ferroportin. The ferroxidase on the placenta is called zyklopen and functions to convert the ferrous iron released by ferroportin to ferric iron, which is then usable by transferrin

The process of iron transport at the placenta is unlike the mechanism seen in the gut (Fig. 2). Unlike transport the gut, uptake of iron into the placenta is exclusively transferrin-mediated. The crucial role of transferrin in placental iron uptake is evidenced by the hypotransferrinemic (hpx) mouse that has a severe transferrin deficiency (Bernstein 1987; Huggenvik et al. 1989; Trenor et al. 2000). In these mice, neonates are severely anemic and survive to only 2 weeks of age unless treated with serum or transferrin (Bernstein 1987; Craven et al. 1987; Dickinson and Connor 1994; Huggenvik et al. 1989; Simpson et al. 1993; Trenor et al. 2000). While the source of iron in the fetus has not been identified, it has been suggested that minimal amounts of transferrin that are present in the mother can function as a source of iron or that an alternative mechanism for NTBI transfer may exist (Trenor et al. 2000). Holo-transferrin has been shown to bind to transferrin receptors on the maternal membrane of the placenta (King 1976; McArdle et al. 1984). The Tf-TfR complex is then endocytosed in a clathrin-coated vesicle (McArdle et al. 1985b; Srai et al. 2002). The total iron binding capacity was shown to be greater in maternal blood than in umbilical cord blood (Okuyama et al. 1985). Thus, the possibility of transferrin transcytosing the placenta would account for only minimal iron transfer across the placenta (Okuyama et al. 1985; Vanderpuye et al. 1986). The mechanism for transferrin-bound iron processing after endocytosis is similar to that of other cell types. Upon endocytosis, the pH decrease in the endosome causes ferric iron to be released from Tf. Ferric iron is reduced to ferrous iron within the endosome, which can then be released from the endosome via DMT-1 (Chong et al. 2005; Georgieff et al. 2000; Gruper et al. 2005; Wong et al. 1987). Cytoplasmic ferrous iron can either be stored by ferritin or incorporated into heme and heme-derivative protein within the placenta or it can be released to the fetal system through ferroportin (Blanck et al. 1983; Brown et al. 1979; Donovan et al. 2000; Hodgson and Juchau 1977; Namkung et al. 1983; Okuyama et al. 1985; Starreveld et al. 1995). Similar to hephaestin in the gut, a ferroxidase, zyklopen, exists in the cellular membrane and functions to oxidize the ferrous iron to ferric iron after transfer through ferroportin (Chen et al. 2010). It is mechanistically important to note here that studies evaluating DMT-1 knockdown suggest that the fetus still receives iron. Mice exhibiting a DMT-1 mutation yielded smaller, iron-deficient pups that were still viable (Gunshin et al. 2005). Additionally, in a study of Belgrade rats, untreated homozygous females were infertile or their pups failed to thrive (Garrick et al. 1997). Together, these studies indicate that DMT-1 loss does not completely eliminate iron transport across the placenta (Garrick et al. 1997; Gunshin et al. 2005). This suggests the possibility of either an additional exporter from the endosome or an alternative, but unidentified, mechanism by which iron is transported. Recently, hypothesized alternative mechanisms include both heme iron transport and transport through channel proteins such as ZIP8 and ZIP14 (McArdle et al. 2014; Nam et al. 2013; Wang et al. 2012). In a recent review, Cao and O’Brien explore the plausibility of placental heme iron transport, but fail to provide direct evidence to suggest that the process occurs (Cao and O’Brien 2013).

### Regulation of iron transport across the placenta

Unlike the regulatory process of iron transfer across the enterocytes of the intestine, regulatory mechanisms of placental iron transfer affect iron status of both the mother and the fetus. The fetal liver functions as the primary director of iron regulation and homeostasis for both the fetus and the mother. Studies first showed that as fetal iron absorption increases during gestation and the maternal iron levels decrease, the number of transferrin receptors on the maternal membrane of the placenta also increases (Gambling et al. 2003; McArdle et al. 1985a). Additionally, it was demonstrated that the majority of placental transferrin receptors were located at the maternal membrane (Bradley et al. 2004). In a series of intricately designed experiments, Gambling et al. demonstrated the ability of the fetal liver to regulate all stages of the maternal iron absorption process (Gambling et al. 2009). They demonstrated that dietary iron availability has direct effects on both maternal and fetal iron status. When fed an iron-deficient diet, the maternal hematocrit was maintained through the first half of the pregnancy, but dropped significantly thereafter (Gambling et al. 2009). Furthermore, the iron-deficient dams had progressively smaller iron stores in the liver from the beginning of term through the end of gestation (Gambling et al. 2009). Inspection of gene expression changes revealed that as the fetal liver iron increased, the maternal liver transferrin receptor mRNA decreased, but only to a certain level (Gambling et al. 2009). This finding indicates that there may be a threshold to the regulatory system whereby the maternal iron status can only become so deficient. Additionally, analysis of hepcidin mRNA correlations demonstrated that the maternal hepcidin expression strongly correlated with fetal iron levels. When fetal liver iron reached a limit, maternal liver hepcidin became upregulated. As expected, placental transferrin receptor decreased as fetal liver iron increased (Gambling et al. 2009; Martin et al. 2004). These data began to provide evidence for the hierarchical nature of placental iron transport in which the fetal iron level is the priority with the maternal hematocrit being the next priority and the maternal iron stores being the last priority. This hierarchy concept provides compelling evidence to suggest a potential benefit of iron supplementation throughout pregnancy to aid in maintaining maternal liver iron stores. Despite the fetal ability to alter placental transferrin receptor, studies also suggest that the placenta itself does not change its rate of iron uptake (Bothwell et al. 1958b; Lane 1968; McArdle and Morgan 1982). These studies were performed with fetectomy, though, which evaluated placental iron accumulation for only a short time period. Because these studies do not account for a potentially longer response time after the loss of a fetal regulatory signal, they can only truly provide information regarding short-term adaptations and therefore may not be a true representation of the overall regulatory system (van Dijk 1988).

When taken together, these studies corroborate early studies on the relationship between fetal and maternal iron status. Most early studies tried to determine the effect of pregnancy on maternal iron absorption and the ability of the mother to compensate for deficiency during pregnancy. It was shown that intestinal iron absorption increased during pregnancy, particularly during the third trimester (Apte and Iyengar 1970; Millard et al. 2004; Svanberg 1975). Millard, et al. also demonstrated increased maternal duodenal expression of DMT-1, Dcytb, and ferroportin (Millard et al. 2004). These increases were coupled with decreases in hepcidin and transferrin receptor expression in the maternal liver (Millard et al. 2004). The alterations in iron transporter expression supports the ability of the mother to respond to pregnancy-induced iron deficiency to increase iron absorption. The benefit of increased iron absorption appears to be species-dependent, however. In the pregnant rat, it was shown that maternal plasma iron turnover increased six-fold during pregnancy, but that maternal gut iron absorption could account for only 40 % of the iron required to meet these demands (Hershko et al. 1976). These data indicate that elevated maternal iron uptake does not meet the requirements of placental iron transfer and maternal iron stores must also be employed in the process. This observation was not supported by studies in the rabbit, where iron is mobilized from stores to a lesser degree (Bothwell et al. 1958b; van Dijk 1988). The discrepancy is further observed in the human where women may range from no significant decreases in iron stores to severe iron deficiency (Wong and Saha 1990). In studies to evaluate iron supplementation, it was shown that a low dose resulted in 72 % of women depleting their iron stores whereas a higher dose results in 54 % of women developing exhausted iron stores (Milman et al. 1991; Thomsen et al. 1993). Despite the varied results seen in mobilization of iron from maternal stores, there is still clear evidence of a maternal iron absorption response to the placental iron transfer depleting maternal hematocrit.

Despite vast evidence regarding the interplay of fetal iron status and placental transferrin receptor levels, little evidence exists regarding the regulation of ferroportin export. A study looking at the effects of maternal iron status showed no significant changes in placental ferroportin expression of mild and moderately anemic mothers (Li et al. 2008). This conflicts with cell culture studies in which the BeWo placental cell line was treated with either the iron chelator desferrioxamine or with holo-transferrin. When treated with the chelator, ferroportin mRNA levels were increased, but the only significant increase in ferroportin protein was at the highest concentration treatment (Li et al. 2012). Ferroportin was less responsive to iron loading. Ferroportin mRNA was only decreased using a high concentration (12.5 mM) of holo-transferrin and protein was only decreased with both the high concentration and after 72 h of treatment (Li et al. 2012). The contradictory results in the case of iron deficiency may be reflective of the immortalized nature of a cell line when compared to primary cells or what is seen in vivo. The cell culture chelation studies do support the Gambling et al. studies previously described in which the placenta responded to iron deficiency and altered its expression profile. It is important to note that while the data were reported as “not shown”, Gambling et al. do state that no changes in total ferroportin expression were observed in their study (Gambling et al. 2009). To date, studies have shown that the fetal liver does produce hepcidin, a peptide known to inhibit ferroportin, proportionally to its iron stores (Gambling et al. 2009). Unfortunately, the effect of this increased fetal hepcidin on placental ferroportin remains unclear—while data suggest no changes in placental ferroportin protein expression, studies have yet to address cellular localization of the ferroportin. If the hepcidin induces ferroportin internalization, but does not lead to degradation, this change in cellular localization would still cause less exporter protein at the fetal membrane.

## Iron transport across the BBB

**Fig. 3:**
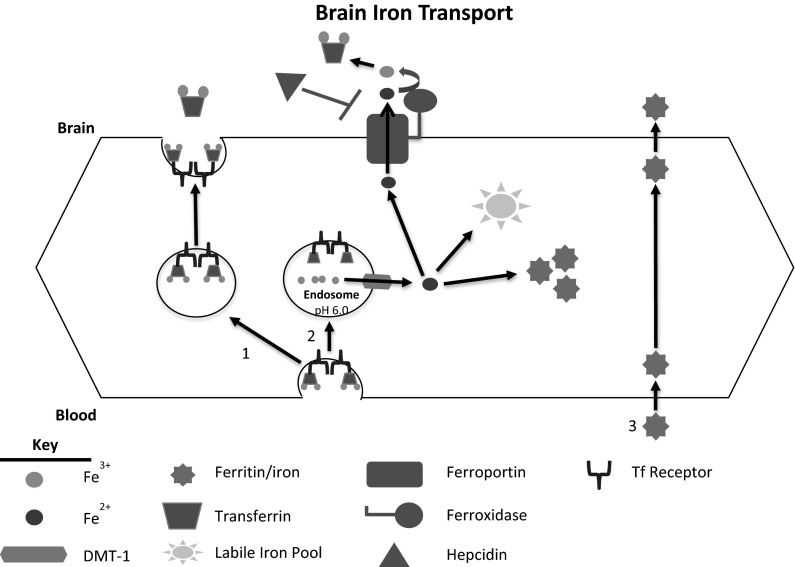
Schematic of brain iron uptake. The process by which iron crosses the BBB has recently been modeled (Simpson et al. 2015). Route *1* represents the currently accepted paradigm of the endothelial cell as a passive conduit in which transferrin binds to its receptor on the luminal membrane, traverses the cell and is deposited into the brain. Route *2* represents the more realistic and data-based model in which transferrin is endocytosed after binding to transferrin receptor. The iron is then released by transferrin within the endosome and transported into the cytoplasm through DMT-1. The intracellular iron can be stored in ferritin or it can be released into the brain through ferroportin. This model accounts for the iron needs of the endothelial cells. Route *3* depicts a potential mechanism by which ferritin can transport iron across the BBB. The possibility of this mechanism has been demonstrated, but further study is required to better understand it

The BBB is composed of the microvasculature of the brain. It is characterized by tight junction formation between endothelial cells and complex mechanisms for influx and efflux that tightly regulate the transport of molecules from the blood to the parenchyma of the brain (Abbott 2002; Abbott et al. 2010; Brightman and Reese 1969; Butt et al. 1990). Regulating iron uptake at the BBB is of great importance because too little or too much iron has been associated with various neurological diseases (Table 1) (Allen et al. 2001; Connor et al. 1995). Knowledge of the exact mechanism for brain iron uptake was stagnant due to the acceptance of the transferrin transcytosis paradigm established nearly 30 years ago (Fig. 3, route 1). This model has been favored by those attempting to use transferrin or antibodies to transferrin receptors as a means to deliver therapeutic compounds to the brain (Cabezon et al. 2015; Pardridge et al. 1991; Shin et al. 1995; Walus et al. 1996; Yoshikawa and Pardridge 1992). Transferrin transcytosis was first demonstrated in vivo by evaluating distributions of ^125^I-Transferrin in the brain and its isolated microvasculature. This study demonstrated that while labeled transferrin remained within the microvasculature, there was also intact transferrin present in the brain supernatant (Fishman et al. 1987). The presence of intact transferrin reaching the brain supports the idea that transferrin, upon endocytosis by the brain microvasculature, is delivered intact into the brain. Subsequent studies demonstrated that radiolabeled OX-26, an antibody to transferrin receptor, is capable of binding brain microvasculature and crossing the BBB into the brain parenchyma in vivo (Friden et al. 1991; Pardridge et al. 1991). Furthermore, it was shown that when OX-26 was conjugated to proteins, more protein traversed the barrier than when the protein was not conjugated (Shin et al. 1995; Walus et al. 1996; Yoshikawa and Pardridge 1992). Of note, all of these studies demonstrate retention in the vasculature in addition to detection in the brain.

**Table 1:** Brain disorders associated with brain iron accumulation or brain iron deficiency

Brain iron accumulation	Brain iron deficiency
Neurodegeneration with brain iron accumulation (Forni et al. 2008)	Restless legs syndrome (Allen and Earley 2001; Connor et al. 2011)
Hemochromatosis (Bartzokis et al. 2010)	Hypomyelination (Beard and Connor 2003)
Amyotrophic lateral sclerosis (Oba et al. 1993)	
Parkinson’s disease (Connor et al. 1995)	
Alzheimer’s disease (Connor et al. 1995)	
Huntington’s disease (Zecca et al. 2004)

Subsequent studies, however, have revealed that iron movement into the brain is not solely via transcytosis. Indeed, the purely transcytosis paradigm for brain iron delivery fails to address a number of basic biological functions of endothelial cells. First, studies showed that when unlabeled transferrin or serum were injected in the presence of ^125^I-transferrin, the amount of labeled protein seen in the brain fraction decreased (Skarlatos et al. 1995). The decreased uptake of ^125^I-Transferrin indicates that this pathway is saturable and dependent upon both serum transferrin levels and the number of transferrin receptors present on the luminal membrane of the endothelium. This observation would suggest no regulation of iron uptake into the brain from the brain side. Moreover, holo-transferrin tightly binds its receptor at physiological pH, which begs the question—if a transferrin iron complex is transcytosed across the endothelial cell, how is transferrin or its iron released from the receptor upon reaching the abluminal surface (Klausner et al. 1983). It has been speculated that a slight environmental pH drop at the abluminal membrane may allow for detachment but no direct evidence supporting these hypotheses has been published (Moos and Morgan 2004) and the pH at which iron begins to dissociate from transferrin is ~5.5 (Dautry-Varsat et al. 1983; Tsunoo and Sussman 1983). Most importantly, the transferrin transcytosis model fails to account for the iron needs of the endothelial cells and does not provide a mode of regulation for iron acquisition by these cells. Given the importance of iron for brain function and the levels of regulation for iron uptake in the gut and placenta it is difficult to explain or understand how the brain would rely on a simple transcyotic method of iron delivery.

### BBB iron uptake mechanism

The most commonly accepted model of brain iron uptake involves transferrin binding to transferrin receptor at the luminal membrane of the endothelial cell (Fig. 3). The Tf-TfR complex is then endocytosed, at which point opinions differ on the fate of the complex and the iron. The widely established transcytosis mechanism asserts that the Tf-TfR complex, with iron bound to transferrin traverses the cell in the endocystosed vesicle. Tf-Fe is then released into the parenchyma at the abluminal membrane. The data for this model, however, are unsatisfactory in that they fail to address such questions as how the transferrin is released from its receptor at the abluminal membrane or more importantly how the endothelial cells comprising the BBB would obtain iron. While there is in vitro evidence for direct transcytosis of transferrin-bound iron, there is also evidence of release of NTBI indicating that an additional mechanism to transcytosis must be occurring (Burdo et al. 2003). The in vitro data suggesting a disconnect between transferrin and iron transfer support in vivo studies in which ^59^Fe-^125^I-transferrin was injected intravenously into rats. These studies demonstrated the presence of non-transferrin bound ^59^Fe in the post-capillary fraction, which represented the brain parenchyma after removal of the microvasculature (Moos and Morgan 1998a). The same studies also demonstrated ^125^I-transferrin in the post-capillary fraction which could reflect direct transcytosis but it is also possible that this transferrin was taken up through the choroid plexus and distributed into the brain parenchyma. The presence of non-transferrin bound ^59^Fe indicates a dissociation of the ^59^Fe from the ^125^I-transferrin within the endothelial cell before it reached the brain parenchyma which is the model we have put forth (Moos and Morgan 1998a).^1^ Pinocytosis remains another potential mechanism that could explain the transport of transferrin across the BBB. Pinocytosis has been demonstrated at the BBB, though less frequently than in other microvessels of the body (Baldo et al. 2014; Coomber and Stewart 1986; Smith and Gumbleton 2006; Strazielle and Ghersi-Egea 2013; Zhao et al. 2014). Several studies have, however, shown that inhibiting micropinocytosis does not affect the transport of either transferrin or H-Ferritin across an in vitro BBB model (Burdo et al. 2003; Fisher et al. 2007). While these studies indicate that pinocytosis does not result in transport of transferrin or H-Ferritin across the BBB, the inhibitor used only inhibits micropinocytosis and does not, therefore, rule out the possibility of low level pinocytosis contributing to transferrin transport at the BBB.

The alternative mechanistic model for brain iron uptake that our laboratory has proposed is that of an endocytic pathway (Fig. 3, route 2). In this proposed model, upon being endocytosed, the lowered pH of the endosome will cause the release of Fe^3+^ from transferrin, which can be reduced to Fe^2+^ by a H-ATPase. The Fe^2+^ is subsequently released into the cytoplasm via DMT-1 where it can be used by the endothelial cell, stored in ferritin, or released into the brain through ferroportin. Upon release, the presence of a ferroxidase such as ceruloplasmin or hephaestin can oxidize the Fe^2+^ and the Fe^3+^ can then be incorporated into apo- transferrin circulating in the brain (McCarthy and Kosman  2013, 2014). One key point of contention with this proposed mechanism is the presence of DMT-1 in the endothelial cells of the BBB. Until recently, there were conflicting studies indicating both the presence and absence of DMT-1 mRNA and protein (Enerson and Drewes 2006; Gunshin et al. 1997; Moos et al. 2006; Siddappa et al. 2002, 2003; Skjorringe et al. 2015). The importance of DMT-1 has been demonstrated physiologically in brain iron uptake by the Belgrade rats, which have a mutation that results in non-functioning DMT-1. Belgrade rats have decreased levels of brain iron, which includes decreased levels of microvessel-associated iron (Burdo et al. 1999).

As further support for intracellular release of iron in the endothelial cells forming the BBB are the reports that endothelial cells of the BBB are capable of storing iron (Simpson et al. 2015). Indeed, the expression levels of ferritin in the BBB are as high as that in the brain per unit protein (Burdo et al. 2004). The BBB acting as an iron reservoir provides evidence against transferrin transcytosis, in which the iron would passively traverse the cell. That endothelial cells are capable of taking up iron and accumulating its own labile iron pool is indicative of an alternative pathway occurring, such as the endocytic mechanism described herein. The endocytic mechanism would result in iron being delivered into the cell’s labile iron pool for storage, usage, or for export, subsequently explaining how endothelial cells could obtain iron and corroborating the DMT-1 mutation effects on iron transport described previously.

Evidence of alternative mechanisms exists for iron uptake at the level of the BBB, but requires vetting before they are included as actual mechanisms for iron transport across the BBB. While most studies have focused on transferrin-bound iron, both NTBI and ferritin-bound iron have importance in brain iron uptake. Studies performed in the transferrin-deficient (hpx) mouse, suggest an alternative to the transferrin-dependent mechanism of iron transport at the BBB. In these studies, non-detectable levels of plasma transferrin had only a marginal effect on brain iron uptake except in the choroid plexus. (Beard et al. 2005). Another study examined the effect of the hypotransferrinemia on the brain iron of neonatal mice and found that the hpx mouse actually had significantly more iron in the brain than wild type mice (Takeda et al. 2001). These data suggest that iron uptake by the choroid plexus is transferrin dependent and that the redistribution from choroid plexus is transferrin dependent. But, the data also suggest that there is an alternative to transferrin -mediated iron uptake in the brain because of the generally similar values of iron found in the various regions of the brain of the hpx mice to that of wild type.

In vitro studies demonstrated that H-Ferritin not only binds to endothelial cells but also moves across the endothelium of a modeled BBB (Fisher et al. 2007). Fisher et al. also demonstrated the presence of ^59^Fe in the brains of rats that received intravenous injection of ^59^Fe-H-Ferritin (Fisher et al. 2007). Together, these studies suggest a role for H-Ferritin in delivering iron to the brain (Fig. 3, route 3). Recently, brain microvascular endothelial cells were also shown to take up NTBI suggesting there is a ferrous iron importer at the luminal membrane (McCarthy and Kosman 2014). These cell culture studies are supported by an in vivo study in which mice deficient in transferrin were able to accumulate ^59^Fe in the brain after injection of non-transferrin-bound ^59^FeCl_3_ (Malecki et al. 1999). Thus, while transferrin may serve as the primary transporter of iron across the BBB, it seems that the BBB exhibits at least two additional mechanisms by which iron can be taken up into the endothelial cells.

### Regulation of iron transport at the BBB

As a result of our recently published model for brain transport, regulation of iron transport at the BBB is *a bona fide* area of investigation. Several studies have focused on the release of iron from the endothelial cell into the brain particularly, the role that ferroxidases play in iron flux from the endothelium of the BBB. It is known that endothelial cells of the BBB, like the enterocytes of the intestine, express hephaestin (McCarthy and Kosman 2013; Patel and David 1997; Qian et al. 2007; Yang et al. 2011). Furthermore, when hephasetin and ceruloplasmin activity was blocked, iron efflux from endothelial cells was also inhibited as the result of ferroportin internalization (McCarthy and Kosman 2013). It was subsequently demonstrated that the addition of soluble ceruloplasmin to cell culture media could reverse the effect of hephaestin and ceruloplasmin knockdown (McCarthy and Kosman 2013). An additional study demonstrated that soluble ceruloplasmin alone could induce iron release from endothelial cells in vitro (McCarthy and Kosman 2013, 2014). These studies suggest that an exocytoplasmic ferroxidase may be important, if not crucial to regulating the release of iron from the endothelial cells forming the BBB. Of note, a recent study demonstrated that in the case of hephaestin knockout, brain iron was elevated (Jiang et al. 2015). This study, however, failed to isolate the microvasculature from the whole brain in their analyses and so the increases in iron may be representative of iron accumulation within the BBB rather than increased levels of iron being transported into the brain.

In addition to the ferroxidase studies, the impact of hepcidin on release of iron into the brain has become a prominent area of research. Cell culture studies have demonstrated both directly (Simpson et al. 2015) and indirectly (McCarthy and Kosman 2014) that hepcidin can reduce release of iron from the endothelium. Furthermore, in vivo studies revealed that intracerebroventricular injection of hepcidin had a similar effect, resulting in decreased iron uptake into the brain (Du et al. 2015). The exposure to hepcidin resulted in decreased levels of transferrin receptor, DMT-1, and ferroportin in the brain microvasculature, consistent with the known function of hepcidin to inhibit release and cause iron to accumulate in the endothelial cell (Du et al. 2015). The elevated iron within the cell would explain the decreases in both transferrin receptor and DMT-1.

The effect of various iron management proteins and cerebrospinal fluid (CSF) from monkeys have been evaluated in a model of the BBB. When endothelial cells of a BBB in vitro model are exposed to CSF from iron-deficient monkeys, iron release is increased when compared to control CSF (Simpson et al. 2015). Furthermore, when astrocyte-conditioned media was added to the endothelial cells, release was modulated by the iron status of the astrocyte; media from iron-deficient astrocytes increased release of iron from the endothelial cell similarly to the CSF from iron-deficient monkeys (Simpson et al. 2015). The increased release of iron suggests the presence of a signaling peptide that induces release, but the identity of the peptide remains to be determined. In the same studies, it was shown that apo-transferrin could cause release, providing a candidate for identifying the peptide present in both the iron-deficient CSF and the iron-deficient astrocyte-conditioned media (Simpson et al. 2015). Lastly, iron-loaded astrocyte-conditioned media had an opposite effect on endothelial cell iron release (Simpson et al. 2015). This identifies the astrocyte as a potential key modulator of iron release from the BBB. It seems highly likely that the astrocyte, which sits at the interface of the endothelium and extends into the brain parenchyma, could function as an iron sensor for the brain and relay brain iron needs to the BBB through release of signaling peptides.

Evidence of regulation was demonstrated in an analysis of microvasculature from patients with restless legs syndrome (RLS) that revealed decreases in transferrin, transferrin receptor, and H-Ferritin (Connor et al. 2011). This study demonstrates the possibility of misregulation of brain iron transport at the BBB. Because it is known that the brain of a RLS patient is deficient in iron, one would expect the relevant signals for stimulating iron release to be present. Thus, the decrease in transferrin receptor and H-Ferritin indicate not only an iron deficient endothelium, but suggests that the BBB is not responding to typical iron cues to upregulate iron transport. Analysis of iron regulatory proteins (IRP) in the same study further support the concept of regulation at the intracellular level (Connor et al. 2011). The study confirmed the presence of IRP1 mRNA in the brain microvasculature and also demonstrated decreased IRP activity within the RLS microvasculature (Connor et al. 2011). IRPs function to stabilize mRNA of both transferrin receptor and DMT-1 when intracellular iron status is low (Eisenstein 2000). The decreased H-Ferritin observed in RLS microvasculature suggests low intracellular iron status, which confounds the observed decrease in transferrin receptor. Thus, the origin of decreased IRP binding activity in RLS brain microvasculature may provide insights into the cause of brain iron deficiency observed in the diseased RLS brain.

## Importance of the HFE protein in barrier iron transport

HFE is an iron management protein that has received considerable interest over the last decade in neurodegenerative diseases. The mutated HFE gene and its protein product are associated with hereditary hemochromatosis (HH), an autosomal-recessive iron overload condition (Beutler et al. 2001; Connor and Lee 2006). After the HFE gene was identified, two mutations, C282Y and H63D, were linked to HH (Feder et al. 1996). The C282Y gene variant has a lower general population prevalence of 1.9 % compared to 8.1 % for the H63D variant, but the C282Y variant is more commonly associated with HH (Merryweather-Clarke et al. 1997). The wildtype HFE protein functions through its association with both β_2_ microglobulin (β_2_M) and transferrin receptor (Feder et al. 1997, 1998; Waheed et al. 1997). The interaction with β_2_M directs HFE to the cell membrane, allowing for HFE to interact with transferrin receptor (Feder et al. 1997; Waheed et al. 1997). The interaction with transferrin receptor regulates iron uptake by allowing for transferrin receptor to associate with only one Fe_2_-Tf molecule (Feder et al. 1998; Lebron et al. 1999). When mutated, the association of HFE to transferrin receptor is lost, resulting in increased Fe_2_-Tf uptake into cells (Feder et al. 1998; Lebron et al. 1999). Bastin et al. showed HFE staining in the placenta, the crypt cells of the gut, and the brain endothelium, which was supported by additional studies demonstrating the presence of HFE in cortical blood vessels (Bastin et al. 1998; Connor et al. 2001). Because of mounting evidence that the presence of HFE mutations may increase risk or alter the course of many neurodegenerative diseases it became important to understand how the HFE protein is functioning to modulate iron transport at the brain but also other physiological barriers (Altamura and Muckenthaler 2009; Loeffler et al. 1995; Zecca et al. 2004).

Several studies have been performed to understand the role that HFE gene variants play in modulating iron absorption at the gut. The studies, however, have yielded mixed results from which the importance of HFE is difficult to interpret. It was shown that deletion of the HFE gene resulted in increased levels of ^59^Fe absorption as well as an increase in the expression of DMT-1 mRNA in the duodenum (Bahram et al. 1999; Fleming et al. 1999). These data are suggestive of HFE playing a crucial role in regulating iron absorption and it was proposed that HFE participates in systemic iron sensing at the crypt cell. Another study, however, failed to detect changes in DMT-1 protein in a mouse model of iron overload that resembles HH (Canonne-Hergaux et al. 2001). Later human studies revealed that patients with HH (C282Y) did not exhibit decreases in duodenal DMT-1 or ferroportin gene expression when compared to normal controls (Stuart et al. 2003). There is clearly a role for HFE in in maintaining systemic iron levels but the mechanism remains unclear and much of the current research has focused on an apparent misregulation of hepcidin resulting from the mutation in HFE (Goswami and Andrews 2006; Nemeth 2008; Piperno et al. 2007; Schmidt et al. 2008).

The role for HFE in the placenta is even less well-elucidated. Studies to determine HFE localization in the placenta were inconsistent. Earlier work revealed highly prevalent HFE staining on the apical membrane, which is also very transferrin receptor dense (Parkkila et al. 1997). In a later study, however, Bastin et al. demonstrated that HFE was actually more prevalent on the basolateral membrane (Bastin et al. 2006). Furthermore, this study demonstrated colocalization of HFE with ferroportin rather than transferrin receptor, which suggests an alternative role for HFE in the placenta (Bastin et al. 2006). More recently, it was shown that knockout of HFE in the mother resulted in elevated iron status in the fetus as well as increased expression of transferrin receptor, DMT-1, and ferroportin in the placenta, which supports a crucial role for HFE in regulating iron transport across the placenta (Balesaria et al. 2012).

For many years, HFE mutations were not thought to impact the brain because of the BBB. This concept was based on erroneous interpretations of two autopsy-based studies in which it was reported there was more iron in the brain in areas not protected by the BBB (Cammermyer 1947; Sheldon 1935). The studies both reported, which failed to get attention in the literature, that there was more iron also in areas behind the BBB (Cammermyer 1947; Sheldon 1935). Subsequent MRI studies (Berg et al. 2000; Nielsen et al. 1995; Rutgers et al. 2007) and a mouse model of the most abundant HFE gene variant (Nandar et al. 2013) report elevated iron in the brain. Studies have shown that HFE protein does exist in the brain endothelium, but the function has not been determined (Bastin et al. 1998; Connor et al. 2001); although there is no reason to suspect it functions any differently in the BBB than in other cells. Several studies have demonstrated elevated brain iron, modified white matter myelination, and increased risk for developing neurodegenerative disease in the presence of HFE mutations (Berlin et al. 2004; Meadowcroft et al. 2015; Nandar et al. 2013; Pulliam et al. 2003). Thus, understanding if and how HFE alters brain iron uptake may help to address questions regarding elevated brain iron and the subsequent risks for disease in the presence of HFE gene mutations.

## Summary of the similarities of gut and placenta iron uptake mechanisms to BBB iron uptake mechanisms

**Fig. 4:**
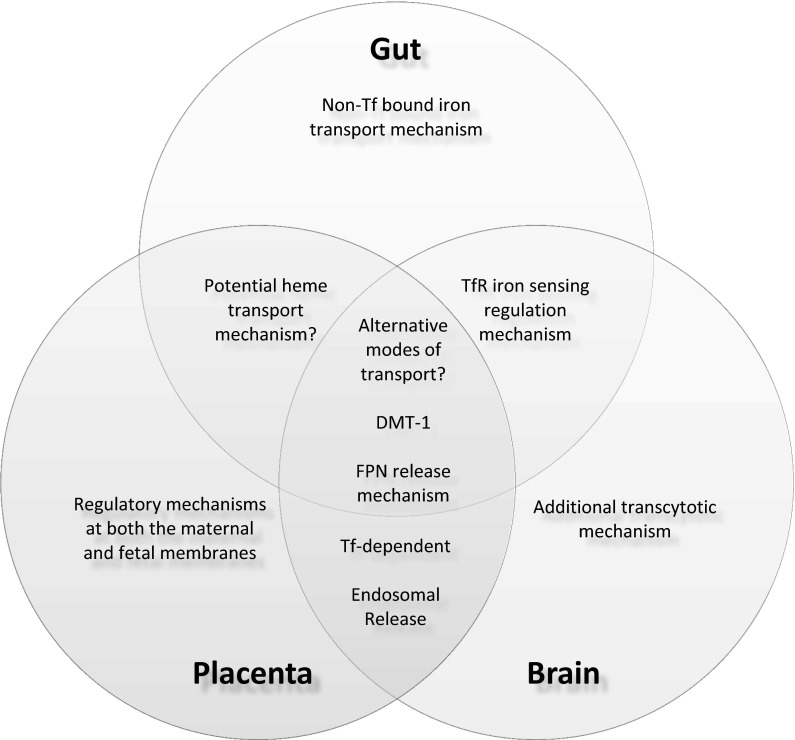
Similarities and differences in iron transport at physiological barriers. When comparing iron transport at the gut, the placenta, and the BBB, there are three key similarities seen at all barriers. First, each barrier appears to demonstrate presently unidentified alternative transport mechanisms. Secondly, DMT-1 is involved in iron trafficking at each barrier. Finally, all three barriers also utilize ferroportin on their basolateral membrane for iron export. Evaluation of each overall mechanism suggests that the BBB is most similar to the placenta. Iron transport at both barriers is primarily transferrin-dependent and involves a mechanism by which iron is released from transferrin in the endosome for export into the intercellular iron pool. Unlike either of the other barriers, the gut requires uptake of non-transferrin bound iron. The BBB appears to be unique in its ability to transcytose transferrin-bound iron. Additionally, the potential for heme transport has been hypothesized in both the placenta and the gut, but there is no evidence to suggest that this occurs at the BBB. In terms of regulation, the BBB demonstrates transferrin–transferrin receptor feedback signaling that is similar to what is observed in the crypt cells of the gut. Meanwhile, the placental regulatory system is unique in that iron transport is under the control of both the mother and the fetus

The purpose of this review was to compare and contrast what is understood about iron transport at other barriers in the body to that in the brain. Current advances in brain iron uptake studies have demonstrated similarities and differences between the roles of iron management proteins as observed in both the intestine and the placenta (Fig. 4). When comparing overall mechanistic schemes, recent BBB studies seem to indicate a higher level of similarity to placental iron transport. Both mechanisms appear to be primarily transferrin-dependent, although the BBB remains distinct in that it evokes both a transcytotic and an endocytic mechanism. Additionally, the endocytic freeing of iron from transferrin and release of ferrous iron into the cytoplasm by DMT-1 appear to be the same at both barriers. However, DMT-1 mutation studies demonstrate a key connection between all three barriers—that additional modes of iron transport must exist in these systems.

Furthermore, the presence of regulatory mechanisms at both the gut and placenta substantiate the hypothesis that regulatory mechanisms exist at the BBB. The BBB shares mechanistic similarities with the placenta for uptake and transport, but regulation of brain iron uptake appears to more closely resemble that of the gut. Transferrin receptors have been demonstrated on both the luminal and abluminal membranes of the BBB (Huwyler and Pardridge 1998; Simpson et al. 2015). The presence of transferrin receptors on the abluminal surface of the BBB could indicate a similar feedback mechanism for regulation as seen in the crypt cells of the intestine. We propose that the transferrin receptors on the abluminal membranes sense iron status in the brain, much like transferrin receptors sense systemic iron status in the gut through the amount of saturated transferrin. If this is the case, during brain iron sufficiency, holo-transferrin could be feeding back into the endothelial cell. The feedback system might result in an increase in intracellular iron, which would decrease luminal transferrin receptor expression and, subsequently, decrease iron uptake.

Moving forward, elucidating the iron transport system at the BBB holds potential significance for the field of drug delivery across the BBB. A long-standing theory has been that the transferrin transyctosis pathway can be exploited to improve drug delivery into the brain (Pardridge et al. 1991; Shin et al. 1995; Walus et al. 1996; Yoshikawa and Pardridge 1992). Unfortunately, this targeting system has been mostly unsuccessful. The approach to using transferrin or antibodies to transferrin receptors to circumvent the BBB for drug delivery failed to consider that transferrin receptor-targeted compounds end up in the late endosome or lysosome rather than being transported directly into the brain (Cabezon et al. 2015). Moving forward, understanding the complete mechanistic and regulatory processes by which iron, and transferrin, cross the BBB and regulation of the pathways for transcytosis versus endocytosis will be of utmost importance in helping to explain a critical nutrient and key factor of multiple brain diseases as well as potentially helping to more effectively deliver drugs to the brain.

## Footnotes

1 One study demonstrated the inability of radiolabeled Tf and iron to penetrate the brain parenchyma when introduced into the ventricular system. This study implemented a single bolus injection in the ventricular system, however, which is not representative of the physiological condition (Moos and Morgan 1998b).
